# 5-Chloro-3-(4-fluoro­phenyl­sulfon­yl)-2,7-dimethyl-1-benzo­furan

**DOI:** 10.1107/S1600536814019114

**Published:** 2014-08-30

**Authors:** Hong Dae Choi, Uk Lee

**Affiliations:** a Department of Chemistry, Dongeui University, San 24 Kaya-dong, Busanjin-gu, Busan 614-714, Republic of Korea; b Department of Chemistry, Pukyong National University, 599-1 Daeyeon 3-dong, Nam-gu, Busan 608-737, Republic of Korea

**Keywords:** crystal structure, benzo­furan, 4-fluoro­phen­yl, C–H⋯O hydrogen bonds, π–π inter­actions

## Abstract

In the title compound, C_16_H_12_ClFO_3_S, the dihedral angle between the plane of the benzo­furan ring system [r.m.s. deviation = 0.007 (1) Å] and that of the 4-fluoro­phenyl ring is 76.11 (5)°. In the crystal, mol­ecules are linked into [010] chains *via* two different inversion-generated pairs of C—H⋯O hydrogen bonds. The crystal structure also exhibits weak π–π inter­actions between the benzene and furan rings of neighbouring mol­ecules [centroid–centroid distance = 3.820 (2) Å].

## Related literature   

For the pharmaceutical properties of compounds containing benzo­furan moieties, see: Aslam *et al.* (2009 ▶); Galal *et al.* (2009 ▶); Howlett *et al.* (1999 ▶); Khan *et al.* (2005 ▶); Ono *et al.* (2002 ▶). For natural products with a benzo­furan ring, see: Akgul & Anil (2003 ▶); Soekamto *et al.* (2003 ▶). For the synthesis of the starting material 5-chloro-3-(4-fluoro­phenyl­sulfan­yl)-2,7-dimethyl-1-benzo­furan, see: Choi *et al.* (1999 ▶). For a related structure, see: Choi *et al.* (2014 ▶).
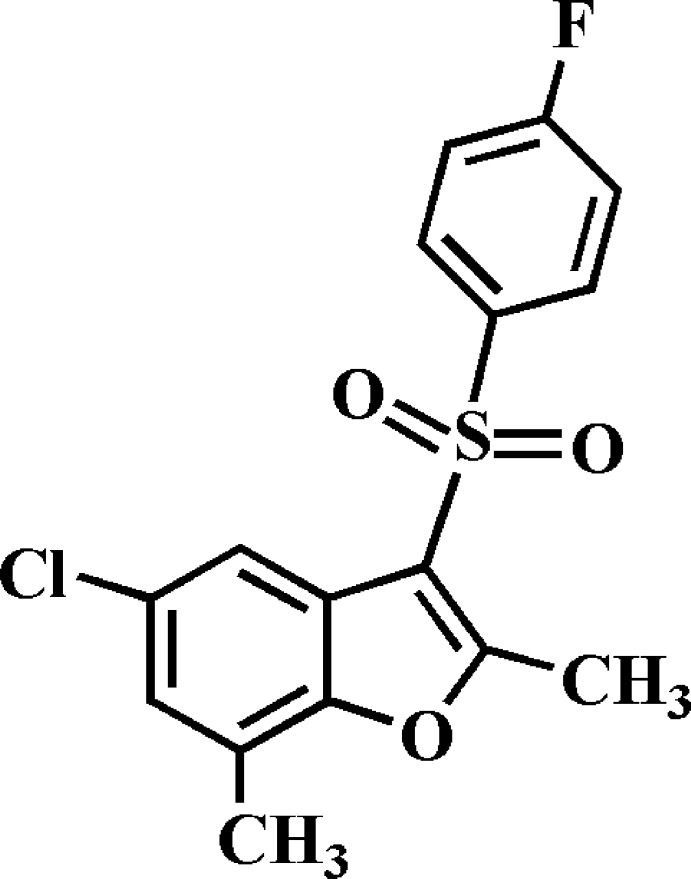


## Experimental   

### Crystal data  

C_16_H_12_ClFO_3_S
*M* *_r_* = 338.77
Triclinic, 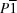

*a* = 8.4338 (3) Å
*b* = 9.9171 (3) Å
*c* = 10.1059 (3) Å
α = 73.988 (2)°
β = 66.155 (2)°
γ = 73.629 (2)°
*V* = 729.02 (4) Å^3^
*Z* = 2
Mo *K*α radiation
μ = 0.43 mm^−1^
*T* = 173 K
0.47 × 0.31 × 0.15 mm

### Data collection  

Bruker SMART APEXII CCD diffractometer
Absorption correction: multi-scan (*SADABS*; Bruker, 2009 ▶) *T*_min_ = 0.825, *T*_max_ = 0.939
13460 measured reflections
3614 independent reflections
3163 reflections with *I* > 2σ(*I*)
*R*_int_ = 0.027

### Refinement  

*R*[*F* ^2^ > 2σ(*F* ^2^)] = 0.037
*wR*(*F* ^2^) = 0.098
*S* = 1.03
3614 reflections
202 parameters
H-atom parameters constrained
Δρ_max_ = 0.34 e Å^−3^
Δρ_min_ = −0.37 e Å^−3^

### 

Data collection: *APEX2* (Bruker, 2009 ▶); cell refinement: *SAINT* (Bruker, 2009 ▶); data reduction: *SAINT*; program(s) used to solve structure: *SHELXS97* (Sheldrick, 2008 ▶); program(s) used to refine structure: *SHELXL97* (Sheldrick, 2008 ▶); molecular graphics: *ORTEP-3 for Windows* (Farrugia, 2012 ▶) and *DIAMOND* (Brandenburg, 1998 ▶); software used to prepare material for publication: *SHELXL97*.

## Supplementary Material

Crystal structure: contains datablock(s) I. DOI: 10.1107/S1600536814019114/mw2126sup1.cif

Structure factors: contains datablock(s) I. DOI: 10.1107/S1600536814019114/mw2126Isup2.hkl

Supporting information file. DOI: 10.1107/S1600536814019114/mw2126Isup3.cml

**Fig. 1.**
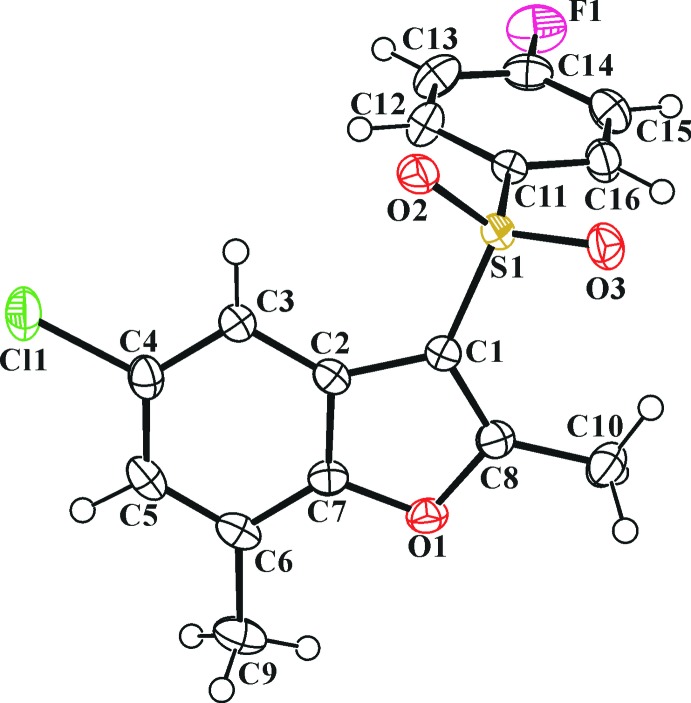
The molecular structure of the title molecule with the atom numbering scheme The displacement ellipsoids are drawn at the 50% probability level. The hydrogen atoms are presented as small spheres of arbitrary radius.

**Fig. 2.**
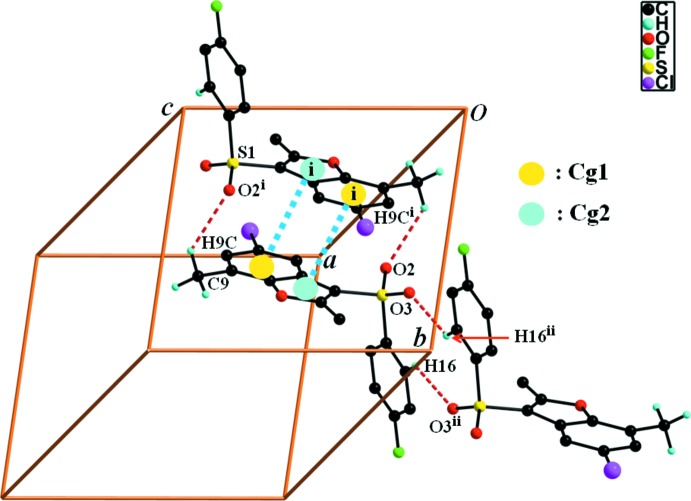
A view of the C—H···O and π–π interactions (dotted lines) in the crystal structure of the title compound. H atoms non-participating in hydrogen-bonding were omitted for clarity. [Symmetry codes: (i) - x, - y + 1, - z + 1; (ii) - x, - y + 2, - z.]

. DOI: 10.1107/S1600536814019114/mw2126fig1.tif
The mol­ecular structure of the title mol­ecule with the atom numbering scheme The displacement ellipsoids are drawn at the 50% probability level. The hydrogen atoms are presented as small spheres of arbitrary radius.

x y z x y z . DOI: 10.1107/S1600536814019114/mw2126fig2.tif
A view of the C—H⋯O and π–π inter­actions (dotted lines) in the crystal structure of the title compound. H atoms non-participating in hydrogen-bonding were omitted for clarity. [Symmetry codes: (i) − *x*, − *y* + 1, − *z* + 1; (ii) − *x*, − *y* + 2, − *z*.]

CCDC reference: 1020842

Additional supporting information:  crystallographic information; 3D view; checkCIF report

## Figures and Tables

**Table 1:** Hydrogen-bond geometry (Å, °)

*D*—H⋯*A*	*D*—H	H⋯*A*	*D*⋯*A*	*D*—H⋯*A*
C9—H9*C*⋯O2^i^	0.98	2.57	3.331 (2)	135
C16—H16⋯O3^ii^	0.95	2.53	3.230 (2)	130

Symmetry codes: (i) 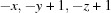; (ii) 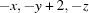.

## supplementary crystallographic information

### S1. Comment

Molecules containing the benzofuran skeleton show interesting pharmacological properties such as antibacterial, antifungal, antitumor, antiviral and antimicrobial activities (Aslam *et al.* 2009, Galal *et al.*, 2009, Khan *et al.*, 2005) as well as being potential inhibitors of β-amyloid aggregation (Howlett *et al.*, 1999, Ono *et al.*, 2002). These benzofuran compounds are widely occurring in nature (Akgul & Anil, 2003, Soekamto *et al.*, 2003). As a part of our ongoing project of 3-arylsulfonyl-5-chloro-2,7-dimethyl-1-benzofuran derivatives containing a 3-methylphenylsulfonyl substituent in the 3-position (Choi *et al.*, 2014), we report herein on the crystal structure of the title compound.

In the title molecule (Fig. 1), the benzofuran unit is essentially planar, with a mean deviation of 0.007 (1) Å from the least-squares plane defined by the nine constituent atoms. The 4-fluorophenyl ring is essentially planar, with a mean deviation of 0.003 (1) Å from the least-squares plane defined by the six constituent atoms. The dihedral angle formed by the benzofuran ring system and the 4-fluorophenyl ring is 76.11 (5)°. In the crystal structure (Fig. 2), molecules are linked *via* pairs of C—H···O hydrogen bonds (Table 1), forming inversion dimers. The crystal packing (Fig. 2) also exhibits weak π–π interactions between the benzene and furan rings of neighbouring molecules, with a Cg1···Cg2^i^ (i: -*x*, 1-*y*, 1-*z*) distance of 3.820 (2) Å and an interplanar distance of 3.641 (2) Å resulting in a slippage of 1.159 (2) Å (Cg1 and Cg2 are the centroids of the C2–C7 benzene ring and the C1/C2/C7/O1/C8 furan ring, respectively),

### S2. Experimental

The starting material 5-chloro-3-(4-fluorophenylsulfanyl)-2,7-dimethyl-1-benzofuran was prepared by the literature method (Choi *et al.*, 1999). 3-Chloroperoxybenzoic acid (77%, 515 mg, 2.3 mmol) was added in small portions to a stirred solution of 5-chloro-3-(4-fluorophenylsulfanyl)-2,7-dimethyl-1-benzofuran (337 mg, 1.1 mmol) in dichloromethane (35 mL) at 273 K. After being stirred at room temperature for 10h, the mixture was washed with saturated sodium bicarbonate solution (2 x 20 mL) and the organic layer was separated, dried over magnesium sulfate, filtered and concentrated at reduced pressure. The residue was purified by column chromatography (hexane–ethyl acetate, 4:1 *v*/*v*) to afford the title compound as a colorless solid [yield 71% (240 mg); m.p. 468–469 K; *R*_f_ = 0.56 (hexane-ethyl acetate, 4:1 *v*/*v*)]. Single crystals suitable for X-ray diffraction were prepared by slow evaporation of a solution of the title compound (30 mg) in ethyl acetate (20 mL) at room temperature.

### S3. Refinement

All H atoms were positioned geometrically and refined using a riding model, with C–H = 0.95 Å for aryl and 0.98 Å for methyl H atoms, *U*_iso_ (H) = 1.2*U*_eq_ (C) for aryl and 1.5*U*_eq_ (C) for methyl H atoms.The positions of methyl hydrogens were optimized using the SHELXL-97 command AFIX 137 (Sheldrick, 2008).

### Crystal data

**Table:**

C_16_H_12_ClFO_3_S	*Z* = 2
*M**_r_* = 338.77	*F*(000) = 348
Triclinic, *P*1	*D*_x_ = 1.543 Mg m^−^^3^
Hall symbol: -P 1	Melting point = 469–468 K
*a* = 8.4338 (3) Å	Mo *K*α radiation, λ = 0.71073 Å
*b* = 9.9171 (3) Å	Cell parameters from 5949 reflections
*c* = 10.1059 (3) Å	θ = 2.2–28.3°
α = 73.988 (2)°	µ = 0.43 mm^−^^1^
β = 66.155 (2)°	*T* = 173 K
γ = 73.629 (2)°	Block, colourless
*V* = 729.02 (4) Å^3^	0.47 × 0.31 × 0.15 mm

### Data collection

**Table:**

Bruker SMART APEXII CCD diffractometer	3614 independent reflections
Radiation source: rotating anode	3163 reflections with *I* > 2σ(*I*)
Graphite multilayer monochromator	*R*_int_ = 0.027
Detector resolution: 10.0 pixels mm^-1^	θ_max_ = 28.3°, θ_min_ = 2.2°
φ and ω scans	*h* = −10→11
Absorption correction: multi-scan (*SADABS*; Bruker, 2009)	*k* = −13→13
*T*_min_ = 0.825, *T*_max_ = 0.939	*l* = −13→13
13460 measured reflections	

### Refinement

**Table:**

Refinement on *F*^2^	Secondary atom site location: difference Fourier map
Least-squares matrix: full	Hydrogen site location: difference Fourier map
*R*[*F*^2^ > 2σ(*F*^2^)] = 0.037	H-atom parameters constrained
*wR*(*F*^2^) = 0.098	*w* = 1/[σ^2^(*F*_o_^2^) + (0.0444*P*)^2^ + 0.4675*P*] where *P* = (*F*_o_^2^ + 2*F*_c_^2^)/3
*S* = 1.03	(Δ/σ)_max_ = 0.001
3614 reflections	Δρ_max_ = 0.34 e Å^−^^3^
202 parameters	Δρ_min_ = −0.37 e Å^−^^3^
0 restraints	Extinction correction: *SHELXL97* (Sheldrick, 2008), Fc^*^=kFc[1+0.001xFc^2^λ^3^/sin(2θ)]^-1/4^
Primary atom site location: structure-invariant direct methods	Extinction coefficient: 0.016 (2)

### Special details

**Table:**

Experimental. 1H NMR (δ p.p.m., CDCl_3_, 400 Hz): 7.98-8.03 (m, 2H), 7.67 (d, J = 2.04 Hz, 1H), 7.17-7.22 (m, 2H), 7.10 (s, 1H), 2.80 (s, 3H), 2.43 (s, 3H).
Geometry. All esds (except the esd in the dihedral angle between two l.s. planes) are estimated using the full covariance matrix. The cell esds are taken into account individually in the estimation of esds in distances, angles and torsion angles; correlations between esds in cell parameters are only used when they are defined by crystal symmetry. An approximate (isotropic) treatment of cell esds is used for estimating esds involving l.s. planes.
Refinement. Refinement of F^2^ against ALL reflections. The weighted R-factor wR and goodness of fit S are based on F^2^, conventional R-factors R are based on F, with F set to zero for negative F^2^. The threshold expression of F^2^ > 2sigma(F^2^) is used only for calculating R-factors(gt) etc. and is not relevant to the choice of reflections for refinement. R-factors based on F^2^ are statistically about twice as large as those based on F, and R- factors based on ALL data will be even larger.

### Fractional atomic coordinates and isotropic or equivalent isotropic displacement parameters (Å^2^)

**Table:**

	*x*	*y*	*z*	*U*_iso_*/*U*_eq_	
Cl1	0.51698 (6)	0.19310 (5)	0.47396 (5)	0.03701 (14)	
S1	0.13342 (5)	0.69006 (4)	0.14183 (4)	0.02143 (12)	
F1	0.70966 (17)	0.98587 (15)	−0.25209 (14)	0.0518 (4)	
O1	0.04838 (15)	0.74576 (13)	0.53640 (13)	0.0263 (3)	
O2	0.18910 (16)	0.54497 (12)	0.11860 (13)	0.0272 (3)	
O3	−0.02442 (16)	0.77606 (13)	0.11823 (13)	0.0291 (3)	
C1	0.1165 (2)	0.68330 (17)	0.32016 (17)	0.0215 (3)	
C2	0.2069 (2)	0.56854 (17)	0.40420 (17)	0.0217 (3)	
C3	0.3177 (2)	0.43625 (17)	0.38227 (18)	0.0235 (3)	
H3	0.3506	0.4013	0.2943	0.028*	
C4	0.3767 (2)	0.35884 (18)	0.49555 (19)	0.0260 (3)	
C5	0.3304 (2)	0.40691 (19)	0.62598 (19)	0.0285 (4)	
H5	0.3761	0.3492	0.6996	0.034*	
C6	0.2188 (2)	0.53740 (19)	0.65043 (18)	0.0267 (4)	
C7	0.1604 (2)	0.61368 (18)	0.53606 (18)	0.0236 (3)	
C8	0.0241 (2)	0.78571 (18)	0.40404 (18)	0.0240 (3)	
C9	0.1674 (3)	0.5919 (2)	0.78961 (19)	0.0361 (4)	
H9A	0.1210	0.6950	0.7737	0.054*	
H9B	0.2713	0.5730	0.8175	0.054*	
H9C	0.0764	0.5431	0.8685	0.054*	
C10	−0.0915 (2)	0.92612 (19)	0.3815 (2)	0.0322 (4)	
H10A	−0.0361	1.0018	0.3790	0.048*	
H10B	−0.2059	0.9286	0.4625	0.048*	
H10C	−0.1092	0.9411	0.2881	0.048*	
C11	0.3084 (2)	0.78021 (17)	0.02639 (17)	0.0224 (3)	
C12	0.4815 (2)	0.70757 (19)	0.0003 (2)	0.0306 (4)	
H12	0.5062	0.6108	0.0468	0.037*	
C13	0.6181 (3)	0.7776 (2)	−0.0943 (2)	0.0374 (4)	
H13	0.7379	0.7301	−0.1138	0.045*	
C14	0.5764 (3)	0.9174 (2)	−0.1594 (2)	0.0339 (4)	
C15	0.4069 (3)	0.9914 (2)	−0.1352 (2)	0.0348 (4)	
H15	0.3834	1.0880	−0.1825	0.042*	
C16	0.2703 (2)	0.92165 (19)	−0.03976 (19)	0.0299 (4)	
H16	0.1511	0.9706	−0.0198	0.036*	

### Atomic displacement parameters (Å^2^)

**Table:**

	*U*^11^	*U*^22^	*U*^33^	*U*^12^	*U*^13^	*U*^23^
Cl1	0.0373 (3)	0.0245 (2)	0.0429 (3)	−0.00018 (18)	−0.0175 (2)	0.00258 (18)
S1	0.0238 (2)	0.0211 (2)	0.01966 (19)	−0.00243 (15)	−0.00994 (15)	−0.00263 (14)
F1	0.0438 (7)	0.0607 (9)	0.0443 (7)	−0.0292 (7)	0.0018 (5)	−0.0068 (6)
O1	0.0261 (6)	0.0302 (6)	0.0231 (6)	−0.0035 (5)	−0.0074 (5)	−0.0100 (5)
O2	0.0366 (7)	0.0233 (6)	0.0244 (6)	−0.0044 (5)	−0.0133 (5)	−0.0062 (5)
O3	0.0253 (6)	0.0319 (7)	0.0299 (6)	−0.0029 (5)	−0.0148 (5)	−0.0005 (5)
C1	0.0220 (7)	0.0222 (8)	0.0199 (7)	−0.0032 (6)	−0.0077 (6)	−0.0042 (6)
C2	0.0229 (7)	0.0233 (8)	0.0186 (7)	−0.0066 (6)	−0.0072 (6)	−0.0015 (6)
C3	0.0250 (8)	0.0225 (8)	0.0216 (8)	−0.0042 (6)	−0.0082 (6)	−0.0025 (6)
C4	0.0252 (8)	0.0218 (8)	0.0285 (8)	−0.0057 (6)	−0.0106 (6)	0.0022 (6)
C5	0.0307 (9)	0.0319 (9)	0.0234 (8)	−0.0122 (7)	−0.0135 (7)	0.0063 (7)
C6	0.0280 (8)	0.0350 (9)	0.0190 (7)	−0.0138 (7)	−0.0076 (6)	−0.0010 (6)
C7	0.0221 (8)	0.0264 (8)	0.0217 (8)	−0.0063 (6)	−0.0053 (6)	−0.0056 (6)
C8	0.0224 (8)	0.0262 (8)	0.0245 (8)	−0.0045 (6)	−0.0086 (6)	−0.0062 (6)
C9	0.0388 (10)	0.0529 (12)	0.0211 (8)	−0.0162 (9)	−0.0100 (7)	−0.0075 (8)
C10	0.0290 (9)	0.0281 (9)	0.0395 (10)	0.0031 (7)	−0.0131 (8)	−0.0134 (8)
C11	0.0243 (8)	0.0240 (8)	0.0188 (7)	−0.0036 (6)	−0.0079 (6)	−0.0042 (6)
C12	0.0262 (9)	0.0245 (9)	0.0385 (10)	0.0002 (7)	−0.0105 (7)	−0.0084 (7)
C13	0.0257 (9)	0.0379 (11)	0.0455 (11)	−0.0041 (8)	−0.0049 (8)	−0.0168 (9)
C14	0.0347 (10)	0.0410 (11)	0.0260 (9)	−0.0166 (8)	−0.0031 (7)	−0.0084 (8)
C15	0.0421 (11)	0.0321 (10)	0.0291 (9)	−0.0124 (8)	−0.0145 (8)	0.0045 (7)
C16	0.0299 (9)	0.0277 (9)	0.0290 (9)	−0.0034 (7)	−0.0131 (7)	0.0012 (7)

### Geometric parameters (Å, º)

**Table:**

Cl1—C4	1.7440 (18)	C6—C9	1.501 (2)
S1—O3	1.4356 (12)	C8—C10	1.477 (2)
S1—O2	1.4361 (12)	C9—H9A	0.9800
S1—C1	1.7332 (16)	C9—H9B	0.9800
S1—C11	1.7648 (16)	C9—H9C	0.9800
F1—C14	1.353 (2)	C10—H10A	0.9800
O1—C8	1.368 (2)	C10—H10B	0.9800
O1—C7	1.382 (2)	C10—H10C	0.9800
C1—C8	1.361 (2)	C11—C16	1.386 (2)
C1—C2	1.448 (2)	C11—C12	1.386 (2)
C2—C7	1.392 (2)	C12—C13	1.385 (3)
C2—C3	1.395 (2)	C12—H12	0.9500
C3—C4	1.382 (2)	C13—C14	1.374 (3)
C3—H3	0.9500	C13—H13	0.9500
C4—C5	1.394 (3)	C14—C15	1.366 (3)
C5—C6	1.387 (3)	C15—C16	1.385 (2)
C5—H5	0.9500	C15—H15	0.9500
C6—C7	1.385 (2)	C16—H16	0.9500
			
O3—S1—O2	119.75 (8)	C6—C9—H9A	109.5
O3—S1—C1	109.59 (8)	C6—C9—H9B	109.5
O2—S1—C1	106.33 (7)	H9A—C9—H9B	109.5
O3—S1—C11	107.27 (8)	C6—C9—H9C	109.5
O2—S1—C11	107.36 (8)	H9A—C9—H9C	109.5
C1—S1—C11	105.71 (8)	H9B—C9—H9C	109.5
C8—O1—C7	107.05 (12)	C8—C10—H10A	109.5
C8—C1—C2	107.60 (14)	C8—C10—H10B	109.5
C8—C1—S1	126.93 (13)	H10A—C10—H10B	109.5
C2—C1—S1	125.40 (12)	C8—C10—H10C	109.5
C7—C2—C3	119.54 (15)	H10A—C10—H10C	109.5
C7—C2—C1	104.59 (14)	H10B—C10—H10C	109.5
C3—C2—C1	135.87 (15)	C16—C11—C12	121.04 (16)
C4—C3—C2	116.27 (15)	C16—C11—S1	119.36 (13)
C4—C3—H3	121.9	C12—C11—S1	119.58 (13)
C2—C3—H3	121.9	C13—C12—C11	119.27 (17)
C3—C4—C5	123.25 (16)	C13—C12—H12	120.4
C3—C4—Cl1	118.38 (14)	C11—C12—H12	120.4
C5—C4—Cl1	118.37 (13)	C14—C13—C12	118.39 (18)
C6—C5—C4	121.29 (16)	C14—C13—H13	120.8
C6—C5—H5	119.4	C12—C13—H13	120.8
C4—C5—H5	119.4	F1—C14—C15	118.07 (18)
C7—C6—C5	114.81 (15)	F1—C14—C13	118.47 (18)
C7—C6—C9	122.84 (17)	C15—C14—C13	123.45 (17)
C5—C6—C9	122.34 (16)	C14—C15—C16	118.13 (18)
O1—C7—C6	124.81 (15)	C14—C15—H15	120.9
O1—C7—C2	110.37 (14)	C16—C15—H15	120.9
C6—C7—C2	124.83 (16)	C15—C16—C11	119.71 (17)
C1—C8—O1	110.39 (15)	C15—C16—H16	120.1
C1—C8—C10	133.98 (16)	C11—C16—H16	120.1
O1—C8—C10	115.63 (14)		
			
O3—S1—C1—C8	−29.69 (17)	C1—C2—C7—O1	0.51 (17)
O2—S1—C1—C8	−160.47 (15)	C3—C2—C7—C6	1.2 (2)
C11—S1—C1—C8	85.62 (16)	C1—C2—C7—C6	−179.20 (15)
O3—S1—C1—C2	153.51 (13)	C2—C1—C8—O1	0.14 (18)
O2—S1—C1—C2	22.72 (16)	S1—C1—C8—O1	−177.13 (11)
C11—S1—C1—C2	−91.19 (15)	C2—C1—C8—C10	179.67 (18)
C8—C1—C2—C7	−0.40 (18)	S1—C1—C8—C10	2.4 (3)
S1—C1—C2—C7	176.93 (12)	C7—O1—C8—C1	0.18 (18)
C8—C1—C2—C3	179.10 (17)	C7—O1—C8—C10	−179.45 (14)
S1—C1—C2—C3	−3.6 (3)	O3—S1—C11—C16	13.38 (16)
C7—C2—C3—C4	−1.0 (2)	O2—S1—C11—C16	143.30 (14)
C1—C2—C3—C4	179.57 (17)	C1—S1—C11—C16	−103.50 (15)
C2—C3—C4—C5	0.2 (2)	O3—S1—C11—C12	−164.96 (14)
C2—C3—C4—Cl1	−179.55 (12)	O2—S1—C11—C12	−35.04 (16)
C3—C4—C5—C6	0.6 (3)	C1—S1—C11—C12	78.15 (15)
Cl1—C4—C5—C6	−179.72 (13)	C16—C11—C12—C13	−0.5 (3)
C4—C5—C6—C7	−0.4 (2)	S1—C11—C12—C13	177.85 (14)
C4—C5—C6—C9	−179.76 (16)	C11—C12—C13—C14	−0.2 (3)
C8—O1—C7—C6	179.28 (15)	C12—C13—C14—F1	−179.88 (17)
C8—O1—C7—C2	−0.44 (17)	C12—C13—C14—C15	0.4 (3)
C5—C6—C7—O1	179.86 (14)	F1—C14—C15—C16	−179.67 (17)
C9—C6—C7—O1	−0.8 (3)	C13—C14—C15—C16	0.1 (3)
C5—C6—C7—C2	−0.5 (2)	C14—C15—C16—C11	−0.7 (3)
C9—C6—C7—C2	178.89 (16)	C12—C11—C16—C15	0.9 (3)
C3—C2—C7—O1	−179.08 (13)	S1—C11—C16—C15	−177.40 (14)

### Hydrogen-bond geometry (Å, º)

**Table:**

*D*—H···*A*	*D*—H	H···*A*	*D*···*A*	*D*—H···*A*
C9—H9*C*···O2^i^	0.98	2.57	3.331 (2)	135
C16—H16···O3^ii^	0.95	2.53	3.230 (2)	130

Symmetry codes: (i) −*x*, −*y*+1, −*z*+1; (ii) −*x*, −*y*+2, −*z*.
