# Supplementary figures and images for: 5-Chloro-3-(4-fluoro­phenyl­sulfon­yl)-2,7-dimethyl-1-benzo­furan
Source: Acta Crystallogr Sect E Struct Rep Online. 2014 Aug 30;70(Pt 9):o1065–6. doi: 10.1107/S1600536814019114 (PMC4186078; doi:10.1107/S1600536814019114)

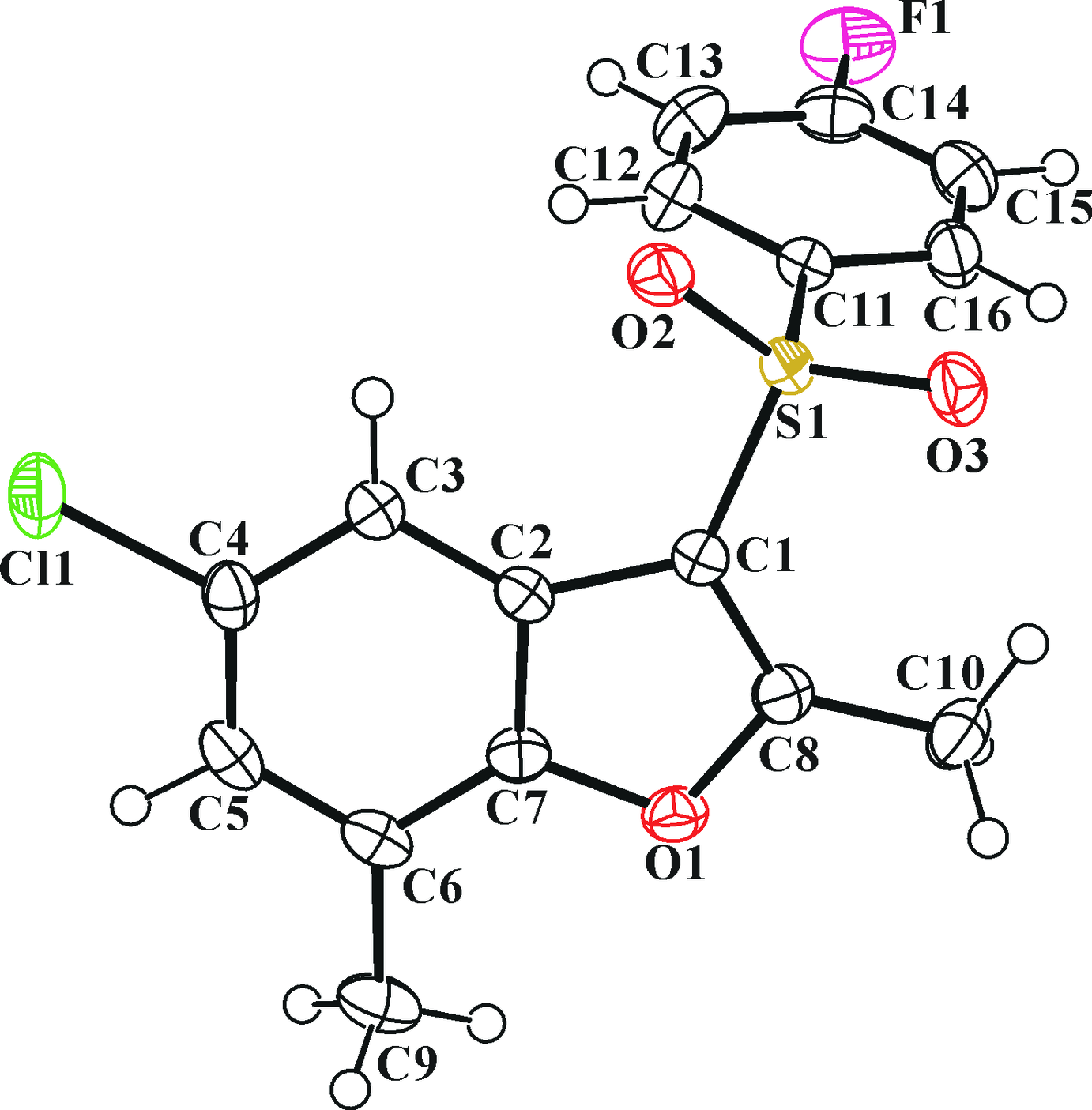

Supplement: Supplementary file 4 [file e-70-o1065-fig1.tif]

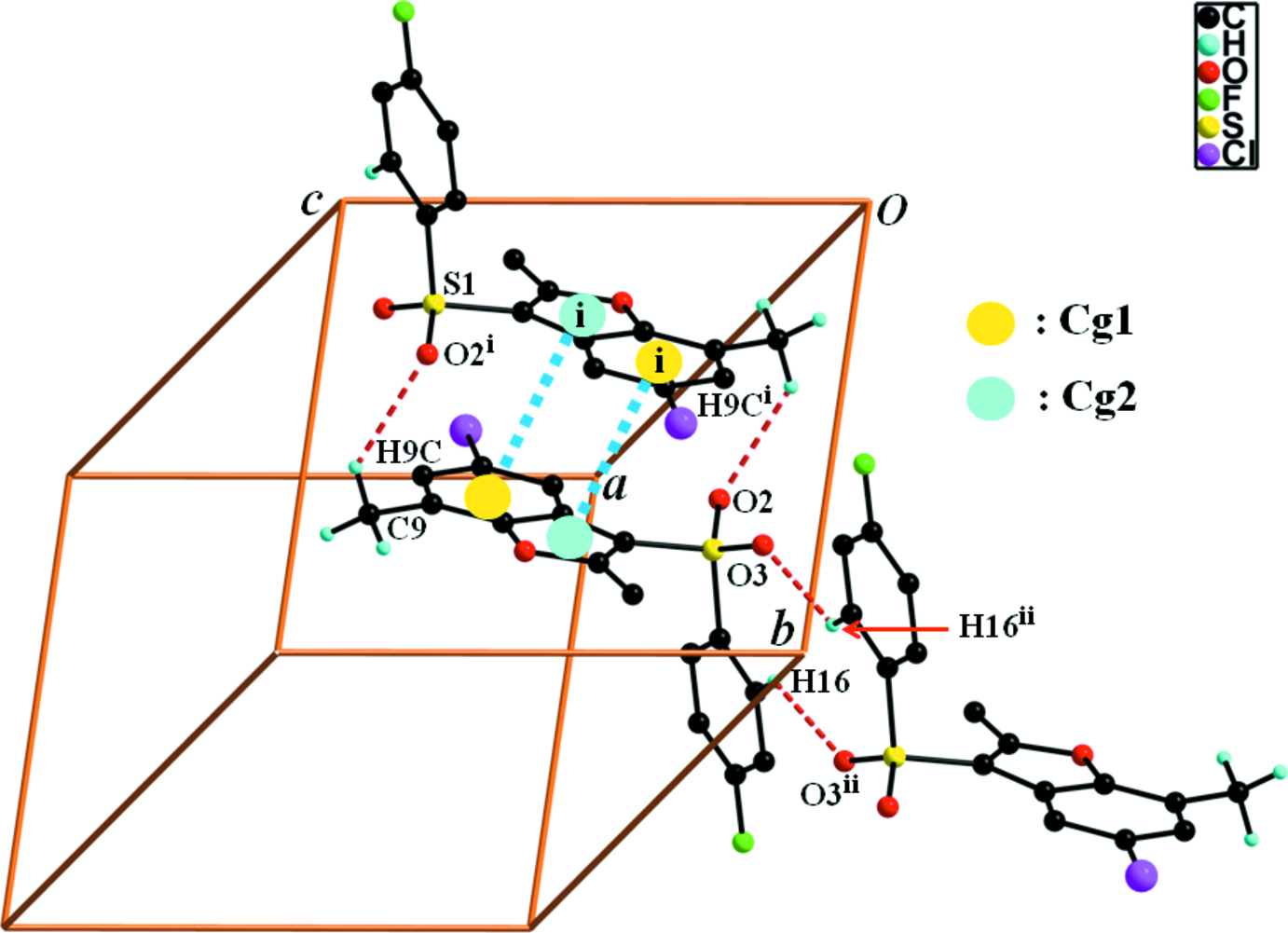

Supplement: Supplementary file 5 [file e-70-o1065-fig2.tif]
